# Effects of Different n6/n3 PUFAs Dietary Ratio on Cardiac Diabetic Neuropathy

**DOI:** 10.3390/nu12092761

**Published:** 2020-09-10

**Authors:** Marjan Urlić, Ivanka Urlić, Hrvoje Urlić, Tomislav Mašek, Benjamin Benzon, Marija Vitlov Uljević, Katarina Vukojević, Natalija Filipović

**Affiliations:** 1 Department of Cardiac Surgery, University Hospital Centre Zagreb, Kišpatićeva 12, 10000 Zagreb, Croatia; urlic.marjan@gmail.com; 2 Department of Oncology, University Hospital Centre Split, Spinčićeva 1, 21000 Split, Croatia; ivanka.drazic@gmail.com; 3 Department of Anatomy, Histology and Embryology, Laboratory for Neurocardiology, University of Split School of Medicine, Šoltanska 2, 21000 Split, Croatia; hrvoje.urlic@gmail.com (H.U.); benzon.benjamin@gmail.com (B.B.); mvitlov@yahoo.com (M.V.U.); kvukojev@gmail.com (K.V.); 4 Department of Animal Nutrition and Dietetics, University of Zagreb Faculty of Veterinary Medicine, 55, 10000 Zagreb, Croatia; tomislav.masek@vef.hr; 5 Department of Anatomy, Histology and Embryology, Laboratory for Early Human Development, University of Split School of Medicine, Šoltanska 2, 21000 Split, Croatia

**Keywords:** diabetes, cardiac innervation, PUFA, autonomic neuropathy

## Abstract

We studied the influence of experimentally induced DM1, in combination with different dietary n6:n3 polyunsaturated fatty acid (PUFA) ratios on different types of nerve fibers in rat myocardium, in order to reveal whether protective/unfavorable effects of different PUFAs on myocardial function in diabetic patients could be a (partial) repercussion of their effect on the changes in cardiac innervation. The control group (c) and diabetic group (stz) were fed with an n6/n3 ratio of ≈7; the diet of the stz+n6 group had an n6/n3 ratio ≈60, while the diet for the stz+DHA group contained 2.5% of fish oil (containing 16% eicosapentaenoic acid—EPA and 19% docosahexaenoic acid—DHA), n6/n3 ratio of ≈1. DM1 was induced by i.p. injection of streptozotocin (55 mg/kg) and rats were euthanized 30 days after induction. Immunohistochemistry was used for the detection and quantification of different types of neuronal fibers in the cardiac septum. We found changes in cardiac innervations characteristics for the initial phase of experimental DM1, which manifested as an increase in total number and area density of all neuronal fibers, measured by Pgp9.5 immunoreactivity. By detailed analysis, we found that this increase consisted mostly of heavy myelinated NF200 immunoreactive fibers and TH immunoreactive sympathetic fibers, while the density of ChAT immunoreactive parasympathetic fibers decreased. In the deep (middle) part of the myocardium, where rare fibers (of all studied types) were found, significant differences were not found. Surprisingly, we found a more consistent protective effect of n6 PUFAs, in comparison to n3 PUFAs supplementation. These results may provide a better understanding of the potential impacts of different PUFA ratios in the diet of diabetic patients on cardiac innervation and genesis and outcome of diabetic autonomic cardiomyopathy.

## 1. Introduction

*Diabetes mellitus* (DM) is a metabolic disease associated with microvascular and macrovascular complications, such as retinopathy, nephropathy, neuropathy, and cardiovascular diseases; whose pathogenesis involves hyperglycemia, insulin resistance, dyslipidemia, hypertension, and immune dysfunction [1]. The metabolic disorders of diabetes lead to diffuse damage of peripheral and autonomic nerves, in addition to the damage of the small vessels [1].

Diabetic cardiomyopathy, whose pathophysiological mechanisms are insufficiently understood, involves damage of contractile cardiomyocytes as well as the autonomic and sensory innervation of the heart, consequently leading to malignant arrhythmias as the most common cause of sudden cardiac death (SCD) [2]. The dysfunction of the autonomic system predicts cardiovascular risk and SCD in patients with type 2 diabetes [3], especially through increased sympathetic activity. Therefore, the modulation of autonomic control may play an important role in the prevention of SCD.

Since many pathological conditions and changes in tissues are associated with long-chain polyunsaturated fatty acid (PUFA) alteration [4,5,6], the beneficial role of the PUFAs consumption and numerous protective effects that long-chain n3 PUFAs exert on the cardiovascular system [7,8,9,10,11] attracted a lot of attention. It is generally considered that a western diet (with high n6/n3 ratio) has unfavorable (including proinflammatory) effects, in contrast to n3 PUFAs which have many protective (and anti-inflammatory) effects on the cardiovascular system generally and subsequently on complications of *diabetes mellitus*. Dietary changes over the past few decades with increasing intake of n6 PUFAs regarding n3 PUFAs show augmentation in the ratio (n6): (n3) PUFA, even up to two to four times (10–20:1) higher than the required, which is considered to be related to a higher incidence of cardiovascular diseases and chronic inflammatory diseases [12].

The effects of PUFAs on the cardiovascular and nervous system have been intensively explored but are not yet explained clearly [13,14,15]. It was found that lean fish (low in fat) consumption by providing higher *n*3 PUFAs in serum and tissues in obese Zucker fa/fa rats might result in benefits for cardiovascular health [16]. In agreement to that, functional and structural abnormalities of cardiomyocytes exposed to high glucose levels, resulting in their hypertrophy, can be prevented by pretreatment with CLA (conjugated linoleic acid) [17]. Moreover, in rats with STZ-induced DM, an improvement of cardiac function due to consumption of n3 PUFAs was observed, measured by increased cardiac output and ejection fraction, as well as stroke volume and stroke work [18]. In addition, in a rat model of metabolic syndrome eicosapentaenoic acid (EPA)/docosahexaenoic acid (DHA) ethyl esters (Omacor) reversed metabolic changes, decreased systolic blood pressure and left ventricular diastolic stiffness, reduced infiltration of inflammatory cells and collagen deposition in the heart, and reduced lipid accumulation and inflammatory cell infiltration [19]. Hence, there is an opinion that n3 PUFAs may protect against SCD [20]. However, some authors pointed out the inconsistency of the positive effects of n3 PUFAs on the cardiovascular system, which is noted through clinical and experimental studies [13]. Among patients with diabetes but without cardiovascular disease, there was no benefit in subjects who received n3 PUFAs compared to those who received a placebo, regarding the incidence of serious vascular events, so routine dietary supplementation with n3 PUFAs is not supported to prevent vascular events, according to this randomized controlled trial [21]. Another meta-analysis found that n3 PUFAs supplementation had no significant association with fatal or nonfatal coronary heart disease or any major vascular events, in people with a history of coronary heart disease [15]. These inconsistencies point to the need for further investigation of the mechanisms of PUFAs effects and their possible side effects [13]. In addition to direct effects on the myocardium, cardiovascular benefits of n3 PUFAs could result also from the reductions in resting heart rate, which is consistent with the effect of augmented baseline parasympathetic tone of the heart [22]. There is proof in animal and in vitro studies that n3 PUFAs have profound antiarrhythmic effects on the heart, which may be mediated by effects of n3 PUFAs on autonomic control or tone. n3 PUFAs (which are present in both nervous tissue and myocardium) may beneficially modulate cardiac autonomic control and possibly reduce the risk of arrhythmias [23]. The limited number of trials that studied the influence of n3 PUFAs on peripheral experimental neuropathy and studies about the regeneration of retinal innervation in diabetic rat models showed their protective effects [24,25,26]. However, despite the fact that many studies have proven the effects of dietary PUFAs on functional changes during cardiovascular complications of DM, no study has explored the effects of dietary PUFAs supplementation on cardiac innervations disturbance caused by *diabetes mellitus*.

Our study hypothesized that protective/unfavorable effects of different PUFAs on myocardial function in diabetic patients could be a (partial) repercussion of their effect on the changes in cardiac innervation. Hence we studied the influence of experimentally induced DM1, in combination with different dietary n6:n3 PUFA ratio on different types of nerve fibers in rat myocardium. Nerve fibers were visualized immunohistochemically, using antibodies against a general neuronal marker, protein gene product 9.5 (PGP 9.5). Tyrosine hydroxylase (TH) was used as a marker for the sympathetic fibers. In addition, neurofilament 200 kDa (NF200; N52) was used to detect sensory heavy myelinated fibers; while antibodies raised against calcitonin gene-related peptide (CGRP) were used to identify the subtype of peptidergic fibers important for the regulation of microcirculation. Finally, antibodies raised against choline acetyltransferase (ChAT) were used as markers of cholinergic (parasympathetic) neuronal fibers.

To the best of our knowledge, this is the first study that explored the effect of different dietary PUFA ratios on changes in cardiac innervations caused by *diabetes mellitus*. These results may provide a better understanding of the potential impacts of different PUFA ratios in the diet of diabetic patients on cardiac innervation and genesis and outcome of diabetic autonomic cardiomyopathy.

## 2. Material and Methods

### 2.1. Ethics

All experimental protocols were approved by the National Ethics Committee and Veterinary Directorate, Ministry of Agriculture (approval number: Kl.01-13/15-08/8 ur.br. 100-01/15-2) Republic of Croatia and conducted according to the European Animal Welfare Act (EP 13/2015).

### 2.2. Experimental Design

A total of 20 male Wistar rats were grown in standard polycarbonate cages in controlled environmental conditions (temperature at 22 ± 1 °C with a 12 h light/dark cycle, with free access to food and water). The standard laboratory diet (containing 20% crude protein, 5% crude fat, and 5% crude fiber) was adjusted with different ratios of n-6/n-3 using different oil blends, consisting of sunflower, fish, and linseed oil, as was published in detail previously [27]. The rats were randomly divided into 4 groups and were given different n6/n3 ratios, according to diet protocol in the study. The control group (c) and diabetic group (stz) were fed with the same protocol with 0.5% of linseed oil and 2% of sunflower oil with a n6/n3 ratio of ≈7. The diet of the stz+n6 group consisted of 2.5% sunflower oil with a n6/n3 ratio ≈60. The diet for the stz+DHA group contained 2.5% of fish oil (containing 16% eicosapentaenoic acid—EPA and 19% docosahexaenoic acid—DHA), n6/n3 ratio of ≈1.

Diabetes was induced in three groups: stz, stz+n6 and stz+DHA, two weeks after the beginning of special diet protocol, by injecting intraperitoneally with 55 mg/kg body weight of streptozotocin dissolved in citrate buffer (pH 4.5) after overnight fasting. The group c received pure citrate buffer solution intraperitoneally, as described previously [28]. The same diet protocol lasted until the end of the experiment. Diabetes was diagnosed if rats had a concentration of blood glucose above 16.5 mmol/L, measured with a glucometer (Accu-Check Go).

The duration of the intervention lasted for 44 days: 14 days before induction of the DM1 and then 30 days after DM1 induction. In our previous works we proved that the same intervention resulted in substantial changes in total fatty acid composition of different tissues, as well as profound changes in fatty acid composition of phospholipids [27,28].

### 2.3. Sample Collection

Thirty (30) days after the streptozotocin/citrate buffer injection, the rats were sacrificed by exsanguination in deep anesthesia (Narketan 80 mg/kg and Xylapan 12 mg/kg; Vétoquinol, Bern, Switzerland). Hearts were extracted and stored in buffered 4% paraformaldehyde for histological analyses. Tissues were dehydrated by ethanol and embedded in paraffin wax as described previously [28]. Paraffin blocks were cut in 5 μm-thick sections and mounted on histological slides.

### 2.4. Immunohistochemistry

Sections were deparaffinized by using xylene and rehydrated by ethanol and water. After deparaffinization, sections were heated for 12 min in citrate buffer (pH 6.0), cooled down and washed in phosphate buffer saline (PBS). Sections were then incubated overnight in the primary antibodies (Table 1). After washing in PBS, an appropriate secondary antibody (Table 1) was applied for 1 h to perform the detection. The sections were washed in PBS. The slides were then air-dried and cover-slipped (ImmuMount, Shandon, Pittsburgh, PA, USA).

### 2.5. Data Acquisition and Analysis

**Figure 1:**
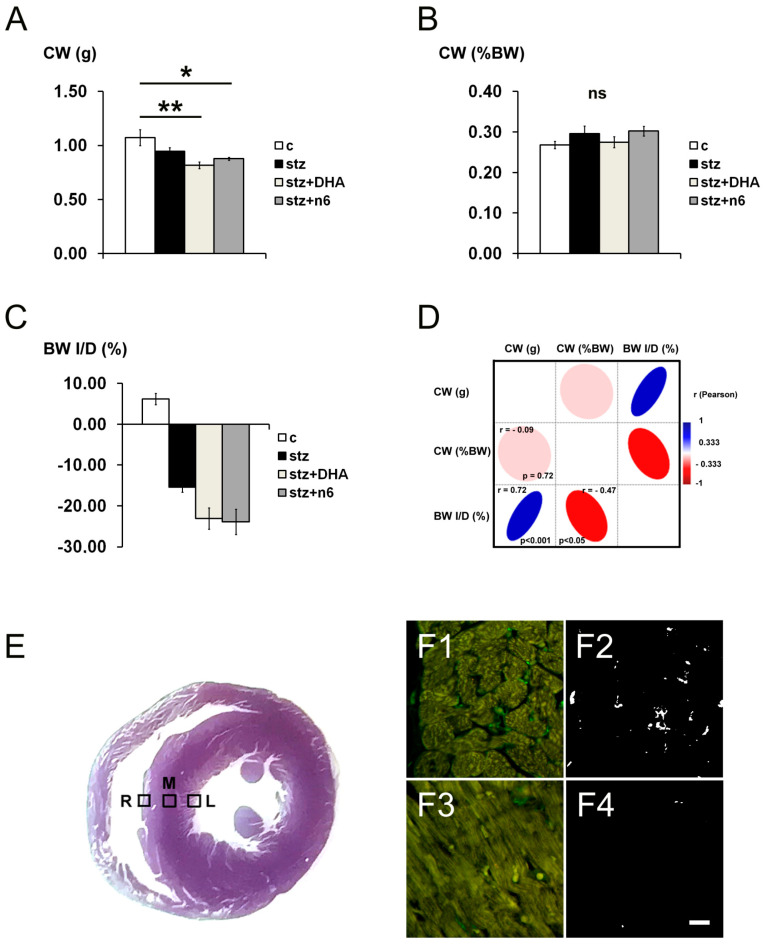
Cardiac weight, body weight changes and sampling of visual fields. (**A**) Cardiac weight (CW; g) of rats in different experimental groups: c—control group; stz—diabetic group fed standard diet (both diets n6/n3 ratio ≈7); stz+DHA—diabetic group supplemented with n3 polyunsaturated fatty acid (PUFA) (2.5% of fish oil, containing 16% eicosapentaenoic acid—EPA and 19% docosahexaenoic acid—DHA; n6/n3 ratio of ≈1); stz+n6—Diabetic group fed with diet that contained 2.5% sunflower oil (n6/n3 ratio ≈60). *—*p* < 0.05; **—*p* < 0.01 difference between indicated groups. (**B**) Cardiac weight expressed as percentage of body weight (%BW). No significant difference in CW expressed as %BW was found (ns). (**C**) Body weight increase or decrease (BW I/D) expressed as % of the initial weight. (**D**) Correlation analysis between CW (g), CW (%BW) and BW I/D (%)—Pearson’s coefficient of correlation (r). (**E**) section through the ventricles of the rat heart (non-related specimen), showing example of positioning of the visual fields for analysis—M—Intramural (Mid.) part of the cardiac septum; R—Right subendocardial area of the septum; L—Left subendocardial area of the septum. (**F**) Representative photomicrographs showing comparison of the density of fibers in the right side of the subendocardial area of the cardiac septum (F1 and F2) and Mid. area (F3 and F4) of the same animal, stained for general neuronal marker Protein g Product (PgP) 9.5 (green). Tresholded figures F2 (tresholded from F1) and F4 (tresholded from F3). Substantially lower density of fibers was found in M, in comparison to R (or L—Not shown). Scale-bar = 20 µm.

Prepared sections were viewed and photographed using a microscope (BX61, Olympus, Tokyo, Japan) and captured using a cooled digital camera (DP71, Olympus, Tokyo, Japan). We used objective UPLFLN40X (Olympus, Tokyo, Japan; magnification 40×; numeric aperture 0.75; working distance 0.51; correction level of chromatic aberration Semiapochromat (FL)). In total, 5–10 visual fields covering subendocardial tissue on both the right-ventricle facing and left-ventricle facing surface of the cardiac septum (Figure 1) were captured. Furthermore, 7–15 visual fields covering the myocardium in the middle layer of the septum were captured. ImageJ Software (National Institutes of Health, Bethesda, MD, USA) was used for further analyses. Figures were processed using Adobe Photoshop 7.0 “Threshold” function whit constant conditions of 25%; In threshold figures, threshold % area was measured using ImageJ, with a restriction of minimally 20 pixels in size for a structure to be considered as a nerve fiber. The nerve fiber area was expressed as a percentage of the cardiac tissue area and as a number of fibers per area unit (only for total fibers marked with PgP9.5).

### 2.6. Statistical Analyses

PAST 3.22 Software was used for statistical analyses [29]. N was 5 per each experimental group. For calculation of the sample size, the Mead’s resource equation was used [30]. The calculated minimal size of the experimental group should be *n* = 4. The normality of distribution was tested by the Shapiro–Wilk test. ANOVA with the Tukey post-hoc test was used to determine significant differences among groups. In the case of deviations from the normal distribution, data were transformed logarithmically prior to the analysis. In the case of unequal variances, ANOVA with Welch correction was used. Statistical significance was set up at *p* < 0.05.

## 3. Results

### 3.1. General Remarks

In the present study, we used different immunohistochemical markers in order to explore the density of innervation in the cardiac septum of diabetic rats in STZ-induced DM1, as well as the influence of different n6/n3 PUFA supplementation. The cardiac weight of diabetic rats decreased in stz+DHA (*p <* 0.01) and stz+n6 groups (*p <* 0.05), in comparison to the control group of rats (Figure 1A). However, when expressed as a percentage of the body weight, there was no significant difference in cardiac weight between groups (Figure 1B), which was related to a substantial decrease in body weight of all three diabetic groups (Figure 1C). We found a strong significant positive correlation between the cardiac weight of rats (in grams) and percentage of change in body weight (increase or decrease from the initial weight) (r = 0.72; *p <* 0.001) and a significant negative correlation between relative cardiac weight expressed as a percentage of body weight and a change in body weight (r = −0.47; *p <* 0.05) (Figure 1D). We explored the density of nerve fibers of the intramural (Mid. area) as well as in the subendocardial part of the septal wall (Figure 1E). Analyzing all types of fibers, we found much denser innervation subendocardially, while the innervation in the Mid. area of the cardiac septum was characterized by rare nerve fibers (Figure 1F), with no significant differences between the experimental groups.

### 3.2. Protein g Product 9.5—Immunoreactive Fibers Density

**Figure 2:**
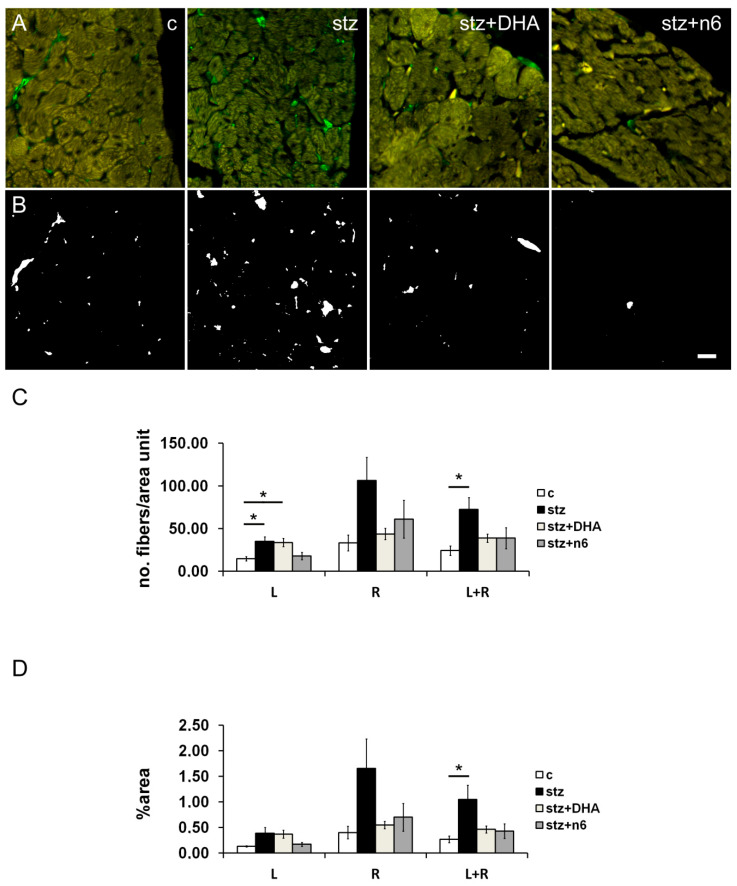
Protein g Product 9.5 immunoreactive nerve fiber density in subendocardial areas of cardiac septum. (**A**) Representative photomicrographs of right side of the subendocardial area of the cardiac septum stained for general neuronal marker PgP 9.5 (green). c—control group; stz—diabetic group fed standard diet (both diets n6/n3 ratio ≈7); stz+DHA—diabetic group supplemented with n3 PUFA (2.5% of fish oil, containing 16% eicosapentaenoic acid—EPA and 19% docosahexaenoic acid—DHA; n6/n3 ratio of ≈1); stz+n6—Diabetic group fed with diet that contained 2.5% sunflower oil (n6/n3 ratio ≈60). (**B**) Threshold figures from A; white—nerve fiber area. (**C**) Neuronal density expressed as number of fibers per area unit. L—Subendocardial area facing the left ventricle; R—Subendocardial area facing right ventricle; L+R—Average from L and R. (**D**) —Neuronal density expressed as percentage of the tissue area. *—*p* < 0.05 difference between indicated groups. Scale-bar = 20 µm.

In the STZ induced diabetic group we found a statistically significant (*p <* 0.05) increase in density of neuronal fibers immunoreactive for PgP 9.5, in comparison to the control group, observed in the average of subendocardial tissue on both the right-ventricle facing and left-ventricle facing surface of the cardiac septum. In the groups with special diets: stz+DHA group and stz+n6 group, there was no detectable change of neuronal fibers PgP. A statistically significant increase in the number of neuronal fibers PgP was also found in stz, in comparison to c group in the subendocardial area facing the left ventricle, as well as on average, for the right and left side of the septum (both *p <* 0.05). In addition, on the left side, a similar increase in PgP 9.5 immunoreactive fibers was also found in the stz+DHA group, when compared to the c-group (*p <* 0.05) (Figure 2).

### 3.3. Neurofilament 200—Immunoreactive Fibers Density

**Figure 3:**
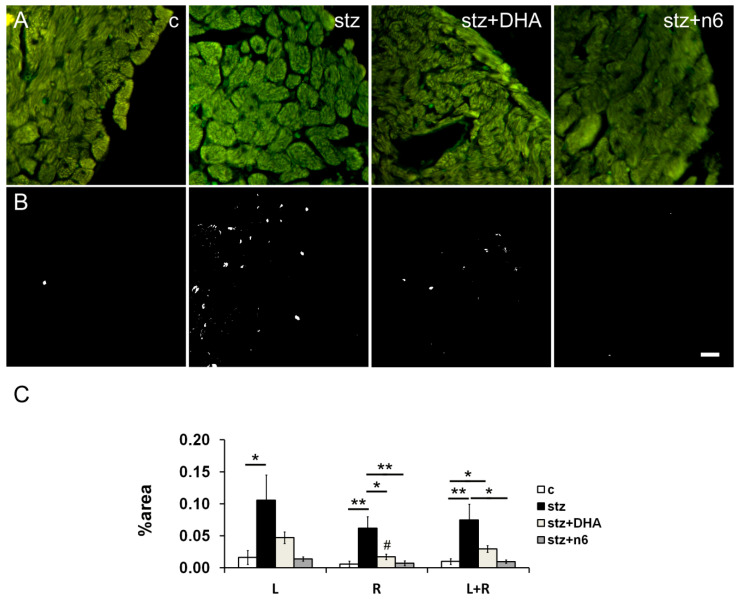
Neurofilament 200 immunoreactive nerve fiber density in subendocardial areas of cardiac septum. (**A**) Representative photomicrographs of right side of the subendocardial area of the cardiac septum stained for NF200, a marker for heavy myelinated sensory fibers (green). C—control group; stz—diabetic group fed standard diet (both diets n6/n3 ratio ≈7); stz+DHA—diabetic group supplemented with n3 PUFA (2.5% of fish oil, containing 16% eicosapentaenoic acid—EPA and 19% docosahexaenoic acid—DHA; n6/n3 ratio of ≈1); stz+n6—Diabetic group fed with diet that contained 2.5% sunflower oil (n6/n3 ratio ≈60). (**B**) Threshold figures from A; white—Nerve fiber area. (**C**) —Neuronal density expressed as percentage of the tissue area. *—*p* < 0.05; **—*p* < 0.01 difference between indicated groups. Scale-bar = 20 µm.

In the stz group, we found a statistically significant increase in the density of NF200 immunoreactive neuronal fibers, when compared to the control group in the subendocardial area facing left (*p* < 0.05) and right ventricle (*p* < 0.01). In the right ventricle facing area, n3 (stz+DHA group; *p* < 0.05) as well as n6 supplementation (stz+n6 group; *p* < 0.01) restoration of the NF200-innervation, resulted in a significant decrease of NF200 density in comparison to diabetic (stz) group. However, on average (R+L), a density of NF200 fibers was higher in stz+N6 (*p* < 0.01), but also in stz+DHA group in comparison to the control (*p* < 0.05); and n6 supplementation resulted in a decrease of NF200 density, in comparison to the stz group (*p* < 0.05; Figure 3).

### 3.4. Calcitonin Gene-Related Peptide—Immunoreactive Fibers Density

**Figure 4:**
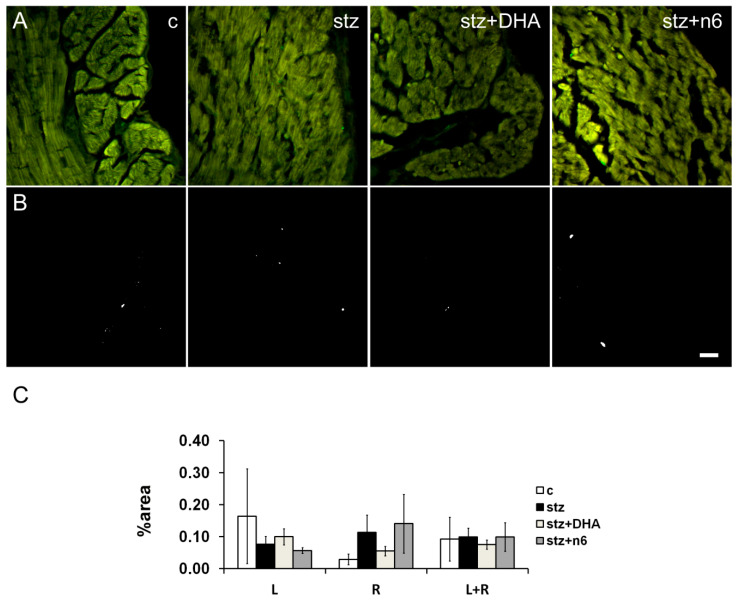
Calcitonin gene related peptide immunoreactive nerve fiber density in subendocardial areas of cardiac septum. (**A**) Representative photomicrographs of right side of the subendocardial area of the cardiac septum stained for neuropeptide CGRP (green). c—Control group; stz—Diabetic group fed standard diet (both diets n6/n3 ratio ≈7); stz+DHA—diabetic group supplemented with n3 PUFA (2.5% of fish oil, containing 16% eicosapentaenoic acid—EPA and 19% docosahexaenoic acid—DHA; n6/n3 ratio of ≈1); stz+n6—Diabetic group fed with diet that contained 2.5% sunflower oil (n6/n3 ratio ≈60). (**B**) Threshold figures from A; white—nerve fiber area. (**C**)—Neuronal density expressed as percentage of the tissue area. Scale-bar = 20 µm.

During the present study, we did not find a statistically significant change in density of CGRP- immunoreactive fibers caused by diabetes alone, nor in combination with different n6/n3 PUFA dietary ratios (Figure 4).

### 3.5. Tyrosine Hydroxylase—Immunoreactive Fibers Density

**Figure 5:**
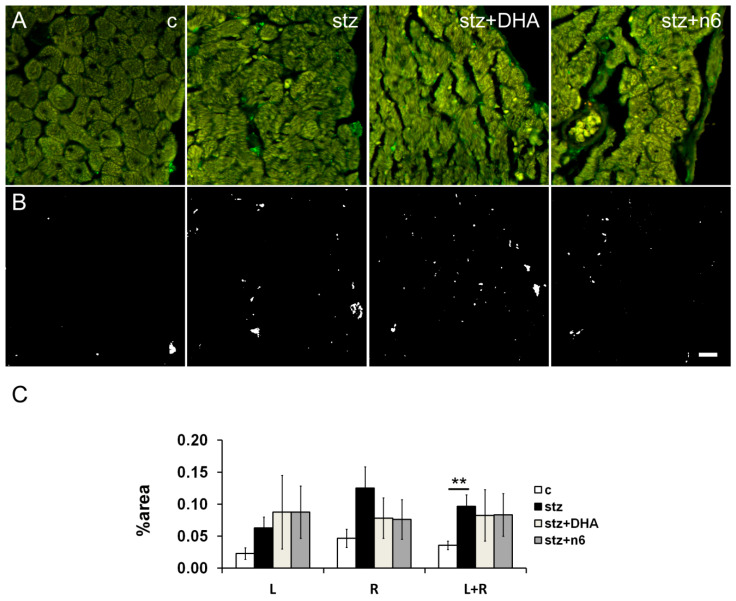
Tyrosine hydroxylase immunoreactive nerve fiber density in subendocardial areas of the cardiac septum. (**A**) Representative photomicrographs of right side of the subendocardial area of the cardiac septum stained for TH, a marker for sympathetic neuronal fibers (green). c—control group; stz—diabetic group fed standard diet (both diets n6/n3 ratio ≈7); stz+DHA—diabetic group supplemented with n3 PUFA (2.5% of fish oil, containing 16% eicosapentaenoic acid—EPA and 19% docosahexaenoic acid—DHA; n6/n3 ratio of ≈1); stz+n6—diabetic group fed with diet that contained 2.5% sunflower oil (n6/n3 ratio ≈60). (**B**) Threshold figures from A; white—nerve fiber area. (**C**)—Neuronal density expressed as percentage of the tissue area. **—*p* < 0.01 difference between indicated groups. Scale-bar = 20 µm.

In the STZ induced diabetic group of rats, we found an increase in the density of TH-immunoreactive neuronal fibers, in comparison to the control group, that was significant in the average of the subendocardial area facing right and left ventricle (*p* < 0.01). Supplementation with any, n3 (stz+DHA group) or n6 (stz+n6 group) did not restore the density of TH-immunoreactive fibers to the control level (Figure 5).

### 3.6. Choline Acetyltransferase—Immunoreactive Fibers Density

**Figure 6:**
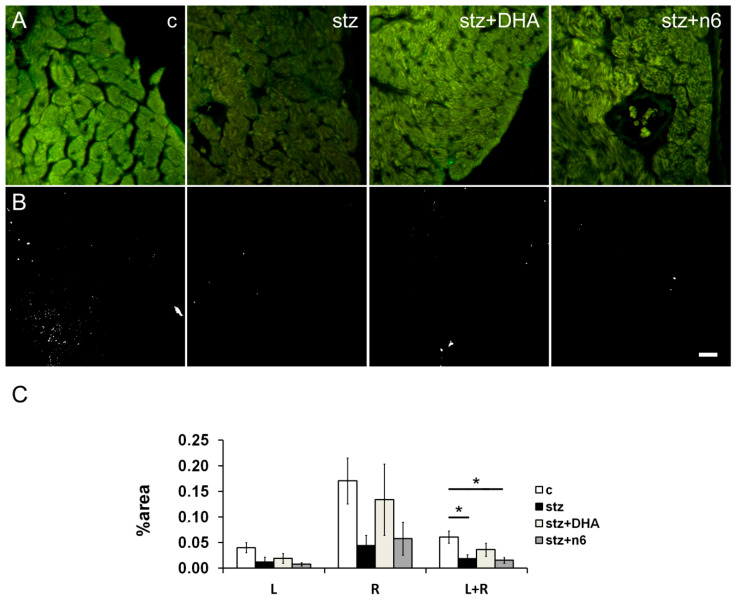
Choline acetyl transferase immunoreactive nerve fiber density in subendocardial areas of cardiac septum. (**A**) Representative photomicrographs of right side of the subendocardial area of the cardiac septum stained for ChAT, a marker for parasympathetic fibers (green). c—Control group; stz—diabetic group fed standard diet (both diets n6/n3 ratio ≈7); stz+DHA—Diabetic group supplemented with n3 PUFA (2.5% of fish oil, containing 16% eicosapentaenoic acid—EPA and 19% docosahexaenoic acid—DHA; n6/n3 ratio of ≈1); stz+n6—diabetic group fed with diet that contained 2.5% sunflower oil (n6/n3 ratio ≈60). (**B**) Threshold figures from A; white—Nerve fiber area. (**C**)—Neuronal density expressed as percentage of the tissue area. *—*p* < 0.05 difference between indicated groups. Scale-bar = 20 µm.

We found a decrease in the density of ChAT-immunoreactive neuronal fibers in the cardiac septum of the STZ induced diabetic group of rats, in comparison to the control group, that was significant in the average of the subendocardial area facing right and left ventricle (*p* < 0.05). Supplementation to any, n3 (stz+DHA group) or n6 (stz+n6 group) did not restore the density of ChAT-immunoreactive fibers to the control level. Moreover, in stz+n6 group density was significantly lower in comparison to the control group (*p <* 0.05; Figure 6).

## 4. Discussion

As previously proven, changes in sensory and sympathetic innervation density in rat hearts with different stages of experimental DM occur [31,32]. The effects of PUFAs on cardiovascular and nervous systems have been intensively explored, but many inconsistencies about the effects of n3 and/or n6 PUFAs on the cardiovascular system, noted through clinical and experimental studies, require further investigation of the mechanisms of their effects and possible side-effects [13,14,15]. Although a limited number of trials that studied the influence of n3 PUFAs on peripheral experimental neuropathy and on regeneration of retinal innervation in diabetic rat models showed protective effects [24,25,26], this is the first study on the effects of PUFAs on cardiac autonomic neuropathy. We tried to define changes in cardiac innervation and the effects of different dietary n6/n3 PUFA ratio analyzing different types of neuronal fibers in the interventricular cardiac septum of rats with an STZ-induced DM1 model.

*Diabetes mellitus* in rats resulted in a decrease in body weight, as a result of metabolic disturbance. An increase or decrease in body weight was followed by the accommodation of the heart, whose weight was also smaller, especially in groups supplemented with n3 or n6 PUFAs, in comparison to the control group. Cardiac adjustment to change in body weight is supported by the absence of differences in cardiac weight expressed as a percentage of body weight and a strong positive correlation between cardiac weight and percentage of change in body weight. However, a negative correlation between relative cardiac weight expressed as a percentage of body weight and a change in body weight might indicate that the decrease in body weight was much more rapid than the decrease in cardiac weight. It is also expected that cardiac innervations might adjust to these changes in myocardial mass.

For the detection of all neuronal fibers in the myocardium, we used staining with pan-neuronal marker Protein gene product 9.5/ubiquitin-C-terminal hydrolase 1 (UCHL-1; PgP 9.5). PgP 9.5 was established as an appropriate marker for nerve fiber detection, due to its widespread presentation in neuronal cell bodies and neuroendocrine cells in the central and peripheral nervous system [33]. PgP 9.5 is localized to the cytoplasm of the neuron, being present in neuronal projections, in addition to the cell body. Antibodies against PgP 9.5 are being used as the main diagnostic tool of small fiber neuropathy (SFN), which can be associated with pre-diabetes status [33]. PgP 9.5 has been already previously used as a general neuronal marker in order to identify intracardiac ganglia [34]. In addition, it was also used as a marker for the evaluation of cardiac damage and regeneration after chemical sympathectomy [35]. In our study, we found significant disturbances in both PgP 9.5-immunoreactive neuronal fiber number and surface area in a cardiac septum of diabetic rats. Subendocardially, an increase in number and the neuronal area was found in rats one month after DM1 induction (Figure 2). The increase in density of subendocardial neuronal fibers was not observed in diabetic n6 and n3 PUFAs supplemented rats, indicating the protective role of these fatty acids. In general, changes were not specific for the particular side of the septal endocardial area, but, interestingly, in n3 PUFAs supplemented rats, we found an increase in the number of subendocardial fibers in the area facing left but not in that facing the right ventricle. An increase in nerve density of PgP 9.5 immunoreactive fibers was in agreement with our previous finding [32] two weeks and two months after DM1 induction. However, in the same study septal PgP 9.5 fiber density did not change significantly. Considering that in the study of Bakovic et al. [32] total (subendocardial, as well as intramural) nerve fibers were analyzed, these data are consistent with the fact that we did not find a significant change in the nerve density in myocardium in the middle part of the septum, opposite to the subendocardial area. In general, analyzing all types of fibers, in our study we found much denser innervation subendocardially, opposite to the innervation in the middle of the cardiac septum, where rare nerve fibers were found, with no significant differences between the experimental groups.

In order to more closely characterize changes in cardiac innervations, we used specific markers for different types of neuronal fibers. Neurofilament 200 kDa (N52; NF200) was applied for the identification of myelinated sensory fibers. Neurofilaments are part of a group of intermediate filaments, the main structural components of neuronal cells. They are the key to communication with axonal transporter proteins and proteins related to microtubules [36]. Neurofilaments are a structural pillar for axons and are essential for their radial growth. They also provide structural stability of myelinated axons which influences nerve conduction velocity [37]. Three subunits of neurofilaments are differentiated by molecular weight. N52 (NF200) is the heavy chain with a molecular weight of 200 kDa and is a marker for sensory myelinated neurons, frequently found in mechanoreceptors and proprioreceptors, as well as in a subpopulation of nociceptive neurons [38,39,40,41]. A source of NF200 immunoreactive fibers in the heart is the dorsal root ganglia (DRG) [42] as well as the vagal nodose ganglia projections [43]. During our study, we found the most profound change in nerve density among NF200 immunoreactive neuronal fibers. In addition, there was a significant decrease of NF200 immunoreactive neuronal fibers in the subendocardial tissue of the septum facing right ventricle compared to the tissue facing left ventricle, when n3 PUFAs were added to the diet.

Our previous results have shown an initial increase of the NF200 fiber density 2 weeks after induction of *diabetes mellitus* by streptozocin injection in Sprague–Dawley rats, which was followed by a decrease 12 months after induction [31]. This is also consistent with our results considering that we found a significant increase in NF200-immunoreactive fibers in the STZ induced diabetic group compared to the control group in the subendocardial tissue of the septum facing right ventricle. Moreover, in the same part of the septum, dietary supplementation with both, n3 or n6 PUFAs resulted in a significant decrease in the amount of NF200 immunoreactive fibers, when compared to the non-supplemented diabetic rats. These results point to the protective role of both, n3 and n6 PUFAs on the damage of heavily myelinated nerve fibers in rat myocardium.

Calcitonin gene-related peptide (CGRP) is a 37-amino acid neuropeptide, primarily localized to C and Aδ sensory fibers, which are represented extensively throughout body innervation, localized voluminously perivasculary, with extension from the adventitia to the media muscle layers of blood vessels. It has a higher localization in arterial than venous tissues. Via its binding to a calcitonin receptor-like receptor (CLR), CGRP functions as a powerful microvascular vasodilator in the coronary circulation, brain and kidneys, having10 times higher power than prostaglandins and 10–100 times greater than other vasodilators such as acetylcholine (ACh) and substance P (SP). It acts protectively on the cardiovascular system and wound healing. In sensory peripheral neuropathy, which is one of the complications of *diabetes mellitus*, a loss of CGRP-containing sensory neurons is found, arising consequently due to the down-regulation of neuronal growth factor (NGF) and manifesting as a loss of nociceptive sensation, also being associated with the poor wound healing. It is possible that the loss of neuronal CGRP impairs cardiovascular diseases in diabetic patients, as there has been shown lower circulating CGRP levels in *diabetes mellitus* patients who suffer from heart disease too [44]. However, in the present study, we did not find significant changes in the amount of the CGRP-immunoreactive neuronal fibers one month after induction of type 1 DM in rats, nor the influence of PUFAs dietary supplementation. These results might indicate that unmyelinated C and Aδ sensory neurons might be less sensitive to change in dietary PUFA content, in comparison to heavy myelinated NF200—Immunoreactive neurons.

Tyrosine hydroxylase (TH) immunoreactivity is frequently used as a marker for sympathetic innervation. TH is the rate-limiting enzyme that synthesizes norepinephrine (NE) from tyrosine, converting it to dihydroxyphenylalanine. NE is a major catecholamine of the sympathetic nervous system that activates β-adrenergic receptors (β-AR). It induces cardiac contractility, acting via cardiac β_1_-AR and β_2_-AR. As one of its complications, DM causes autonomic nerve dysfunction (with the superiority of sympathetic tone) [45,46]. TH is found to be decreased in diabetic mouse and rat heart, while β_1_-AR and β_2_-AR are inactivated/reduced and down-regulated, consequently leading to decreased contractility of the heart, which can arise due to an increased sympathetic-excitation in the diabetic heart [47]. It has been considered that an increase in ventricular arrhythmia inducibility in long term diabetic rats might be related to sympathetic-parasympathetic imbalance and relative sympathetic hyperinnervation (detectable by measuring TH-positive nerve fibers and the corresponding mRNA expression levels in the proximal and distal regions of the left ventricle) [48]. It was found that sympathetic nerve remodeling predominates during 10-weeks of STZ-induced diabetes [45]. In our previous study, we found the largest increase of TH fiber density in the rat heart 2 months after induction of the DM1 model, with the beginning of the proliferation of sympathetic nerve terminals already 2 weeks after induction of DM and there was no change detected in the later stages of DM (6 and 12 months) [31]. This is consistent with our finding of a significant increase of TH fibers density in the subendocardial area one month after DM1 induction. However, we did not find the effect of a diet with different ratios of PUFAs on changes in sympathetic innervation.

Choline acetyltransferase (ChAT, EC2.3.1.6) is the enzyme responsible for the biosynthesis of neurotransmitter ACh. It has been used as a specific indicator for the function of cholinergic neurons in the central and peripheral nervous system, as it is selectively synthesized in the perikaryon of cholinergic neurons and transported to the nerve terminals [49]. Previous studies pointed to the parasympathetic myocardial denervation (proven by immunohistochemistry, using ChAT as a marker), which was concomitant with sympathetic denervation, in STZ-induced diabetic rats 3 months, and even more pronounced 6 months after diabetes induction. These changes might result in exacerbation of parasympathetic/sympathetic disbalance, which can possibly enhance the risk of ventricular arrhythmia. These results are in accordance with the pattern of neural damage noticed in humans [48,50,51,52]. In agreement, autonomic nerve remodeling of the rat heart was noted already in the first 3 weeks after STZ application, with a decrease of parasympathetic tone [45]. Moreover, after 10 weeks, parasympathetic nerves showed more injury and also weaker recovery than sympathetic nerves [45]. In addition, changes in cardiac parasympathetic innervations that manifested through a reduction in cardiac ganglion cell size and number were observed already 4, 8, and 12 weeks after STZ-induction of diabetes in rats [53]. The results of our study are consistent with all the above mentioned since we also found a decrease in the amount of ChAT-immunoreactive fibers in the myocardium of diabetic rats when compared to the control group. It was the same for the diabetic group fed with n6 PUFAs supplemented diet. However, in the group of diabetic rats that were receiving n3 PUFAs supplemented diet the decrease was smaller, and it was not significant.

In summary, we found changes in cardiac innervations characteristic for the initial phase of experimental DM1, which manifested as an increase in total number and area density of all neuronal fibers, measured by Pgp9.5 immunoreactivity. By detailed analysis, we found that this increase consisted mostly of heavy myelinated NF200 immunoreactive fibers and TH immunoreactive sympathetic fibers; while the density of ChAT immunoreactive parasympathetic fibers actually decreased. All of these changes were in agreement with the previous data, with the exception of data for CGRP immunoreactive fibers that did not change significantly during our present study. However, it is important to mention that these changes were found only in the subendocardial area of the septal myocardium, where the highest density of all types of fibers was observed. On the contrary, in the deep (middle) part of the myocardium, where rare fibers (of all studied types) were found, significant differences were not found.

Observed increase in subendocardial nerve fiber density could be considered as a sign of pathological nerve sprouting, which is already previously characterized and attributed mostly to a change in nerve growth factors in diabetic tissue. It could affect generating and conducting an electrical impulse in myocardium and cause arrhythmia (especially in case of TH immunoreactive sympathetic fibers), affecting the sensory input from myocardium (in case of sensory NF200 immunoreactive fibers) but also influencing blood flow regulation.

Surprisingly, we found a more consistent protective effect of n6 PUFAs, in comparison to n3 PUFAs supplementation. Namely, in n6 PUFAs supplemented diabetic rats, an increase in total PgP9.5 immunoreactive fibers was not observed in any part of the myocardium and a significant difference in comparison to diabetic (non-supplemented) rats was found for the right side of the subendocardial area. On the other hand, although there was no change totally (concerning right and left subendocardial area), on the left side an increase in density of total PgP9.5 fibers similar to that one in non-supplemented diabetic rats was found, which consisted mostly from the increase in NF200 immunoreactive fibers. Neither one of the dietary treatments, n3 nor n6 supplementation, reversed the increase of sympathetic TH-immunoreactive- or decreased the parasympathetic ChAT-immunoreactive fiber density. They also did not have an influence on CGRP-immunoreactive fiber density.

Our results are partially in agreement with a recent study that used a rat model for late-stage type 2 diabetes. Enrichment of the diet with menhaden oil (20:5/22:6 n3 EPA/DHA) and flaxseed oil (18: 3 n-3, alpha-linolenic acid) corrected peripheral nerve damage caused by diabetes [54], and authors concluded that long-chain n6 and n3 PUFAs could be a beneficial treatment for diabetic peripheral neuropathy. One of the mechanisms of the menhaden oil on its own was the relaxation of the epineurial arterioles of the sciatic nerve [54]. Similarly, as in the mentioned study, the results of our study could be explained by several mechanisms. Besides the influence on the neural circulation [54,55], restoration of Na, K-ATPase activity [56,57] and Nerve Growth Factor (NGF) tissue expression [58], as well as the increased insulin sensitivity [59] and the wide spectrum of anti-inflammatory effects [60,61], could contribute to the neuroprotective effects of PUFAs, especially n3 [62,63].

However, the protective effect of n6 PUFAs, which we found to be more consistent for a group of myelinated NF200 immunoreactive fibers might be explained by the fact that they make a large proportion of myelin fatty acid composition. In rats, n6 PUFAs make more than 14 percent of all fatty acids composing myelin and 12.5 percent of brain ethanolamine glycerophospholipids (EPG), a major constituent of myelin, while n3 PUFAs constitute only 3.7 myelin fatty acids, both being highly dependent on the dietary fatty acid intake [64]. In the mouse brain, EPG contain more than 30 percent of n6 PUFAs, being also larger than the content of n3 (n3/n6 ratio = 0.77) [65]. During DM, a dramatic disturbance of the central and peripheral nervous system fatty acid metabolism and composition occurs [4,66,67,68,69], but dietary fatty acid supplementation could prevent these disturbances [27]. Larger needs for myelin composition preservation could be an explanation for the more persistent effect of n6, in comparison to n3 PUFAs, in the prevention of myelinated sensory fibers disturbance that we found in the hearts of diabetic rats during the present study. The dietary intervention that we used was a combined effect of prevention and a reversal model, which has relevance for healthy lifestyle effects on diabetic complications in general. However, further studies are needed in order to separate possible preventive from therapeutic effects of PUFA.

The main limit of the study is that we used only immunohistochemistry as an analytical method. However, results of the western blot analysis would show only an amount of a certain protein in the tissue, but they would not show anything about the distribution of the certain protein, or whether the protein of interest was present in particular fibers or some other cells. They also would not reveal the morphology, number, density, or appearance of the particular neuronal fibers. In addition, a western blot analysis might overestimate the amount of protein, especially neuropeptides (CGRP) that might be spilled-out in the tissue, hence results do not show a number, morphology, and potential sprouting of neuronal fibers. On the other hand, PCR might underestimate the amount of a factor, since some of them are not produced in the neuronal fibers. We used immunohistochemistry since it is a method of choice in most of the comparable studies on peripheral diabetic neuropathy, including cardiac neuropathy, and it is also a standard method in diagnostics of the peripheral neuropathy.

DM1 represents only a smaller proportion of patients with diabetes and autonomic neuropathy. However, concerning the overall number of diabetic patients, this proportion still contains a large number of patients, who suffer from serious diabetic consequences and complications, including diabetic autonomic neuropathy and cardiovascular disease. Tissue damage in these patients is often more rapid and extensive, due to a higher level of glycaemia. Although a recent study [70] has shown preservation of cardiac sympathetic function in DM1, this might be the result of rather selective inclusion criteria for diabetic subjects, since only patients with no signs of microvascular complications were included, while the patients with a history of cardiovascular disease were excluded from the study. That particular experimental design enabled selective following of the association between sympathetic nerve function, oxidative metabolism, resting blood flow, left ventricle efficiency, and function in healthy diabetics, as was stated by the authors themselves. Hence, we should compare with cautions these results to pathological condition of the DM1 model, whose purpose was to evaluate an influence of DM1 on cardiac nerve pathology in diseased rats and then to see potential effects of different n6/n3 PUFA ratios on this pathology. We used a streptozotocin-induced DM1 model that enables clear separation of effects of dietary intervention with a different PUFA ratio from effects of a high-fat diet, which is usually used (alone or in combination with streptozotocin) to cause the DM2 model. Although maybe of lesser clinical relevance than the DM2 model (due to lesser proportion, but still large number of patients with DM1), the used model DM1 may give us valuable information about the influence of dietary intervention on diabetic autonomic neuropathy, that might be an initial point for further studies that will use genetic models for both DM1 and DM2.

The study conducted by Stevens and collaborators [71] has shown proximal sympathetic hyperinnervation in diabetic patients with diabetic autonomic neuropathy with distal denervation. This is in agreement with our finding of an increase in proximal septal TH-innervation in DM1-model-rats. However, Stevens et al. did not measure the parameters of innervation for other types of nerve fibers in the heart (for instance NF200+, CGRP+, or Chat+). We focused on the innervation in the proximal part of the septum since this area is representative and comparable to the other research studies. A septum is important because it contains parts of the cardiac conductive system, which is essential functionally and under the influence of neuronal input. However, future studies would be needed in order to evaluate the effects of PUFA on cardiac autonomic innervation in other parts of the myocardium, including distal parts of the septum.

## 5. Conclusions

Despite the many studies concerning cardiac functional changes related to the dietary supplementation with n3 PUFAs during diabetes, this is the first study exploring the effects of different dietary n6:n3 PUFAs ratios on changes in cardiac nerve fibers caused by *diabetes mellitus*. Results of the present study may imply that possible changes in the everyday diet of patients with *diabetes mellitus* have a substantial impact on the most fatal complication of DM. These results may lead us to a better understanding of the potential influence of different dietary PUFA ratios on genesis and the outcome of diabetic cardiomyopathy and cardiac autonomic neuropathy.

## Figures and Tables

**Table 1:** Primary and secondary antibodies.

Primary	Antibody	Code no.	Host	Dilution	Source
	PGP9.5 Monoclonal Antibody (BH7)	480012	Mouse	1:500	Invitrogen
	Anti-Neurofilament 200 kDa Antibody, clone N52	MAB5266	Mouse	1:100	Sigma-Aldrich
	Anti-CGRP	ab36001	Goat	1:1000	Abcam
	Anti-Tyrosine Hydroxylase antibody	ab113	Sheep	1:500	Abcam
	Anti-Choline Acetyltransferase Antibody	AB144P	Goat	1:100	Millipore
Secondary	Alexa Fluor@4 88AffiniPure D onkey Anti- Mouse lgG (H+L)	715-545-150	Donkey	1:400	Jackson Immuno Research Laboratories
	Alexa Fluor@4 88 AffiniPure D onkey Anti- Goat lgG (H+L)	705-545-003	Donkey	1:400	Jackson Immuno Research Laboratories
	Donkey Anti-Sheep IgG H+L (Alexa Fluor^®^ 488)	ab150177		1:400	Abcam
